# Comparison between wharton’s jelly and collagen natural hydrogels for human ovarian follicle transplantation as an artificial ovary

**DOI:** 10.1371/journal.pone.0329690

**Published:** 2025-08-14

**Authors:** Farnaz Tajbakhsh, Mohammad Kazemi Ashtiani, Somayeh Tavana, Ashraf Moini, Rouhollah Fathi

**Affiliations:** 1 Department of Developmental Biology, University of Science and Culture, Tehran, Iran; 2 Department of Embryology, Reproductive Biomedicine Research Center, Royan Institute for Reproductive Biomedicine, ACECR, Tehran, Iran; 3 Department of Cell Engineering, Cell Science Research Center, Royan Institute for Stem Cell Biology and Technology, ACECR, Tehran, Iran; 4 Department of Endocrinology and Female Infertility, Reproductive Biomedicine Research Center, Royan Institute for Reproductive Biomedicine, ACECR, Tehran, Iran; 5 Breast Disease Research Center (BDRC), Tehran University of Medical Science, Tehran, Iran; 6 Department of Gynecology and Obstetrics, Arash Women’s Hospital, Tehran University of Medical Sciences, Tehran, Iran; Jinan University, CHINA

## Abstract

Artificial ovary (AO) is a bioengineered approach aimed at increasing fertility potential in certain cases, especially in women and prepubertal girls with a history of cancer. Some natural and synthetic materials can be used for ovary bioengineering. In the current study, we compared two natural hydrogel composites containing alginate (Alg): Wharton’s Jelly (WJ/Alg) and Collagen (Col/Alg) as models for human AO. In total, six experimental groups were designed: WJ/Alg, Col/Alg, WJ/Alg^+FSH^, Col/Alg^+FSH^, WJ/Alg^+EPO^, and Col/Alg^+EPO^ (n = 60). In each group, 40 isolated human ovarian follicles were seeded in 10 µl of the desired hydrogel and xenotransplanted into the right side of the peritoneum in ovariectomized NMRI mice for 1 week. FSH (7.5 IU) and Erythropoietin (EPO; 200 IU/kg as an angiogenic factor) were injected every other day. Histological and immunohistochemical assessments for Vimentin, CD45, and Ki67; Gene expression analysis for *GDF9*, *Vegf,* and *CD45*; and hormonal assays for estradiol and progesterone were performed. Histological staining showed that WJ/Alg can support follicle growth. In contrast, most of follicles in Col/Alg remained at the primordial stage. Although FSH injection helped granulosa cells differentiation, especially in the WJ/Alg group (507.6 ± 134.1 vs 1444 ± 493.6), and EPO injection increased blood vessels in both groups (*p*-value<0.0001), the follicles could not preserve in the experimental groups. In addition, there were no significant differences in gene expression and hormonal assays across all groups. Based on the histological analysis, the WJ/Alg-base artificial ovary provided better support for follicle development after 1 week compared to the other groups. In addition, Adjusting FSH and EPO dosage may improve follicle survival and growth in future studies.

## Introduction

In recent decades, increasing survival rates among cancer patients, along with the adverse effects of cancer treatments, have heightened the need for fertility preservation strategies. The construction of an artificial ovary (AO) is a bioengineering approach to assist prepubertal girls and women whose ovaries are at risk of cancer treatments [1,2], and provide an appropriate environment for follicles and cell survival and growth [3,4]. It provides benefits such as porosity and biodegradability, which promote follicle and cell growth and migration, as well as enhance cell adhesion and the ability to support angiogenesis [5]. In recent years, various natural materials including plasma clot [6,7], collagen [6], alginate [8,9], gelatin [10], or natural extracellular matrices (ECMs) like decellularized ovary [11–13], as well as synthetic materials such as polyethylene glycol (PEG) [14] have been used to provide a suitable niche for three dimensional (3D) follicular culture and transplantation in the form of an artificial ovary. However, bioengineering research on human ovarian tissue is less extensive than that on mouse tissue, possibly due to limited access to human ovarian tissues and challenges associated with xenotransplantation. To date, several matrices including plasma clot [15,16], fibrin [17,18], decellularized ovarian ECM [19], and PEGylated fibrin [20] have been utilized as human ovarian matrix.

Collagen is a major component of ECM in mammalian tissues, and is readily accessible [21]. Collagen-based materials have been used in various forms for a wide range of bioengineering applications, including ovarian tissue engineering [22]. Collagen-based decellularized ECM derived from ovary [11,13,23], amniotic membrane [24] or Collagen-based bioink from mouse ovaries [10] can be considered as a natural material for follicular culture and transplantation, additionally, it can be used as a hydrogel for in vitro and in vivo follicular growth [6,25]. collagen hydrogel scaffold, like other hydrogels is a 3D, water-based construct that can mimic an ECM environment to support cell or follicle growth, proliferation, and differentiation, due to its structural and biological properties [22,26]. However, weak mechanical strength, rapid degradation in physiological environment, high shrinkage, and opacity are factors that can limit the applications of collagen hydrogels [27]. Therefore, it is necessary to crosslink collagen with other materials such as alginate, chitosan, or hyaluronic acid hydrogels to enhance collagen stability [21,22].

Wharton’s Jelly (WJ) is another natural material obtained from the umbilical cord [28]. WJ contains various growth factors such as insulin-like growth factor-1 (IGF-1) and platelet-derived growth factor (PDGF) and it is a rich source of collagen, proteoglycans, and glycosaminoglycans with an ovarian-like structure. The first decellularized WJ hydrogel (dWJh) used as AO was developed at the Royan research institute (Tehran, Iran), and it supported the growth of isolated mouse ovarian follicles both in vitro and in vivo [29]. Subsequently, dWJh was used to construct a human AO in comparison with alginate in our previous unpublished research. The results showed that dWJh supported the growth of human ovarian follicles at the xenografted site. In the current research, WJ was combined with alginate to enhance its physical and biological properties.

On the other side, various limitations, including hypoxia and re-angiogenesis, can affect the outcome of organ transplantation [30]. Some conditions can modulate these challenges. Erythropoietin (EPO) is a principal hematopoietic hormone and pleiotropic growth factor that can increase angiogenesis at transplanted sites [31,32], improve oxygen and nutrient supply in transplanted tissue, as well as enhance cell growth stimulation [33–35]. Additionally, EPO has been reported to exert anti-apoptotic and anti-inflammatory effects, which further contribute to the improved survival and function of transplanted tissues [36].

on the other hand, some factors have a crucial role in ovarian follicle growth. One of them is follicle-stimulating hormone (FSH) [37]. FSH is an endocrine glycoprotein that is released from the pituitary gland. FSH induces follicle growth and maturation due to its vital role in granulosa cell (GC) proliferation and antrum formation. Utilizing gonadotropins can help the initial stages of follicle activation and development [38,39]. Dolmans et al. (2008) used FSH to achieve human antral follicles after 5 months of xenotransplantation [15]. Moreover, the synergistic interaction between FSH and other intraovarian factors, such as insulin-like growth factors (IGFs) and growth differentiation factor-9 (GDF-9), has been shown to further enhance follicular responsiveness and support sustained development [40].

The aim of this research is to compare the functionality of WJ/Alginate (WJ/Alg) and Col/Alginate (Col/Alg) composites as two different natural artificial ovaries in maintaining the survival of xenotransplanted human ovarian follicles for 1 week. In addition, the effects of two very important factors including FSH and EPO were investigated by histological, molecular, and hormonal analyses.

## Methods and materials

### Ethics

This study was approved for the utilization of umbilical cords and human ovarian tissue samples based on the ethical committee of Royan Institute (IR.ACECR.ROYAN.REC.1400.023 ethical ID; https://ethics.research.ac.ir/ProposalCertificateEn.php?id=195875&Print=true&NoPrintHeader=true&NoPrintFooter=true&NoPrintPageBorder=true&LetterPrint=true). This study started on 2021.06.01 and ended on 2024.06.01. All samples, including umbilical cords and human ovarian tissues, were obtained after the individuals signed informed consent forms (They were between 22 and 27 years of age).

### Experimental design

**Fig 1:**
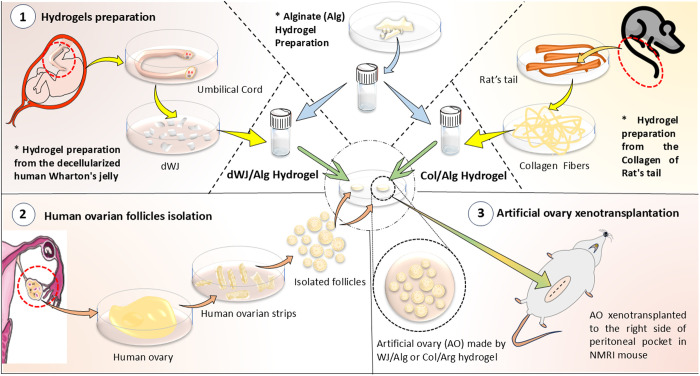
Graphical abstract of study design.

In this interventional research, two different natural hydrogels, including decellularized Wharton’s Jelly/Alginate (dWJ/Alg) composite and Collagen/Alginate (Col/Alg) composite were utilized, and compared in a 3D model of isolated human ovarian follicle xenotransplantation (Fig 1).

In summary, WJ was obtained from the human umbilical cord after cesarean surgery. The dWJ/Alg solution was prepared following the decellularization of WJ. Collagen fibers were derived from rat tail and the collagen hydrogel was prepared. In the next step, 40 partially isolated small human follicles (40–150 µm) were inserted into 10 µl of artificial ovary (dWJ/Alg or Col/Alg). Each artificial ovary was xenotransplanted into a peritoneal pocket in the right side of the abdominal cavity of ovariectomized NMRI mice for 1 week (three biological replicates for each AO). In addition, there were four other groups including dWJ/Alg^+FSH^, Col/Alg^+FSH^, dWJ/Alg^+EPO^ and Col/Alg^+EPO^ that investigated the effect of hormonal (FSH) and angiogenic (EPO) supports on survival and growth in transplanted follicles. Finally, histological, immuno-histochemical, hormonal, and molecular evaluations were performed.

### WJ decellularization

WJ decellularization method was performed according to a previous study with some modifications [29]. In summary, human umbilical cords (UCs) were utilized from 5 healthy women (aged between 25–35 years old) after cesarean surgery and transferred to the laboratory of the Royan Institute in a flask containing a cold (4°C) physiological saline serum medium. In the next step, the vein and arteries were removed and small fragments of WJ were incubated in 10 mM Tris (Sigma-Aldrich, USA) and 0.1% ethylenediaminetetraacetic acid (EDTA; Sigma) (pH = 8) for 16 hours at 4°C for decellularization process. Then, WJ fragments were transferred into a new flask containing 10 mM Base Tris-buffered saline (TBS; Tris-base 2.4 g/100 ml, NaCl 8.8 g/100 ml, HCl 12N 1.3 ml), 0.03% sodium dodecyl sulfate (SDS; Sigma-Aldrich, USA), and 0.1% EDTA (pH = 7.6) on a shaker for 24 hours. In the next step, WJ strips were washed with Tris and then placed into a new flask containing 50 mM Tris-HCl (Sigma-Aldrich, USA) and 10 mM MgCl_2_ (Sigma-Aldrich, USA) for 3 hours (pH = 7.5) on a shaker. Subsequently, WJ fragments were washed 7 times with phosphate-buffered saline (PBS) over 72 hours. DNase I (5 µl/ml; Sinaclon, Iran) and RNase A (10 µl/ml; Sinaclon, Iran) were added into one rinsing solution for 3–4 hours. Then dWJ was lyophilized and chopped.

### Characterization of the decellularized WJ

Histological analysis of 4`,6-diamidino-2-phenylindole (DAPI) and Hematoxylin and Eosin (H&E) staining, and DNA content assessment was utilized to confirm the decellularization process as described below.

### H&E and DAPI staining

The native and decellularized WJ tissues were placed and fixed in a 4% paraformaldehyde solution for at least 24 hours. After paraffin embedding, random sections (thickness = 5 µm) were selected for H&E staining and DAPI staining.

### DNA content

The DNA content was employed to measure the remaining DNA in decellularized tissues [29]. For this purpose, 500 µl of digestion buffer and 20 µl of proteinase K were added to both decellularized and native tissue, vortexed, and incubated at 55°C for 12 hours. Subsequently, 500 µl of phenol-chloroform was added to the solution, and vortexed for 1 minute. The solution was left for 20 minutes at room temperature (RT). Subsequently, it was centrifuged at 13700 rpm for 20 minutes at 4°C. The uppermost layer was carefully transferred to a new microtube. After shaking the microtube, an equal volume of cold ethanol (100%) was added to the medium. The mixture was then centrifuged at 8500 rpm for 15 minutes at 4 °C to remove supernatant. In the next step, 500 µl of cold ethanol (70%) was added and centrifuged at 8500 rpm for 15 minutes at 4°C. The sample was left to dry after removing the supernatant. Finally, to measure the optical density (OD) of each sample by Nanodrop system (Thermo Fisher, USA), 20 µl of H2O was added. The decellularized tissues were compared with native tissues (n = 3).

### Production of dWJ/Alg hydrogel

To make 1.5% dWJ/Alg, 30 mg of dWJ powder was thoroughly stirred with 1 mg of Pepsin and 1 ml of 1 N acetic acid for 24–48 hours. Then, the dWJ solution was neutralized with 0.5 N NaOH (Sigma-Aldrich, USA). Then 500 µl of the dWJ solution was mixed with 500 µl of 1.5% alginate solution in a 1:1 ratio. The final solution was sterilized under UV conditions for future use [29].

### Collagen/Alginate hydrogel preparation

The collagen fibers of five rat tail tendons were collected and washed with PBS. The tendons were immersed in ethanol for 5 minutes to remove fats. Then they were immersed in 70% isopropanol for 5 minutes. The tendons were washed with PBS again and then dissolved in acetic acid (0.02 M; 100 ml for every tail) overnight at 4°C. The solution was centrifuged at 1000 rpm for 30 minutes. The upper solution was transferred to a new petri dish for the freeze-drying process.

For Col/Alg hydrogel preparation, 5 mg of collagen powder was dissolved in 1 ml Acetic acid (0.05 M) and neutralized with NaOH (0.5 M) (pH = 7). Then 750 µl of collagen solution was mixed with 250 µl alginate 3% (the final concentration of collagen was 0.4%). This solution was placed on a stirrer for 3 hours, and it was stored in the refrigerator for the next steps.

### Vitrification of human ovarian tissue

Human ovaries were vitrified based on the protocol of Royan Human Ovarian Tissue Bank (Royan OTB, Tehran, Iran) [41]. Human ovarian tissues (HOT) were obtained from 5 transgenders men (between 22 and 27 years of age) who underwent gender-affirming surgery. The ovarian strips were stored in the liquid nitrogen tank for future research.

### Isolation of human ovarian follicles

Two to four strips of vitrified ovarian tissues were chopped into 0.5 × 0.5 mm pieces by a tissue chopper (McIlwain Tissue Chopper, UK). Then the chopped tissues were transferred into a Falcon tube containing HTCM, Collagenase IA (1 mg/ml; Gibco, USA), and 50 µl/ml Neutral Red (NR; Sigma, Kanagawa, Japan), and placed in the water bath (37°C) for 60 minutes to enzymatical digestion and, gentle agitation were performed every 20 minutes. The enzyme was inactivated with cold HTCM and 20% human serum albumin (HSA). Human ovarian follicles in all stages (primordial (40–60 µm), primary (60–80 µm), and secondary (80–150 µm)) were partially isolated with two insulin syringes and transferred into new droplets using a 130 µm Pasteur pipette [15].

### Artificial ovary construction and xenotransplantation

Approximately 40 viable human follicles were placed in 10 µl of either Col/Alg or dWJ/Alg solution. Then, the 50 µl CaCl2 bath was covered with a solution containing follicles to create artificial ovaries (AOs). AOs were xenotransplanted into the peritoneal pocket on the right side of the NMRI mouse (6–8 weeks) for 1 week. There were 6 xeno-transplanted groups including dWJ/Alg, Col/Alg, dWJ/Alg^+FSH^, Col/Alg^+FSH^, dWJ/Alg^+EPO^, and Col/Alg^+EPO^ with 10 biologicals replicates (5 for histological assessments and 5 for gene expression). Mice were kept under controlled conditions with a 12-hour light/12-hour dark cycle at 20–22°C.

In the surgery process, all NMRI mice were weighed and anesthetized by ketamine and xylazine based on their body weight. The mouse abdominal area was cleaned and shaved under sterile conditions. The abdomen was cut to a 1.5 cm length and the mice ovaries were removed. Every single AO was xenografted into the right side of the intraperitoneal pocket (n = 10 for each AO). The grafted site was fixed with thin, non-absorbable sutures, and the abdominal wall and skin were closed using 6/0 sutures (Teb Keyhan, Karaj, Iran). After 1 week, mice were anesthetized, blood samples were collected for hormonal analysis, and xenotransplanted AOs were retrieved.

Twenty xenotransplanted mice (10 mice in dWJ/Alg^+EPO^ and 10 mice in Col/Alg^+EPO^ groups) received EPO to increase the angiogenesis process and reduce the necrosis at the transplantation site. Two hundred IU/kg EPO was injected subcutaneously three times (days 0, 2 and 5) during a week. On the other side, 10 mice in dWJ/Alg^+FSH^ group and 10 mice in Col/Alg^+FSH^ group received 7.5 IU FSH every other day (days 0, 2 and 5) for a week. All injections were performed subcutaneously.

### Histological and immunohistochemistry (IHC) analyses

One week after xenotransplantation, the AOs (n = 5 for each group) were fixed in 4% paraformaldehyde and tissue processing was performed. Prepared paraffin blocks were serially sectioned (5 µm thickness) for H&E and IHC staining (n = 30) [29].

The fifth section of all serial sections were placed on poly-L-lysine-coated slides for subsequent immunostaining. Immunohistochemistry analyses were done with Rabbit Anti-CD45 Antibody [EP322Y] (ab40763), Rabbit Anti-Vimentin Antibody [EPR3776] (cytoskeleton) (ab92547), and Rabbit Anti-Human Ki-67 Monoclonal Antibody [Clone SP6] (MAD-000310QD-3). The inhibition of endogenous peroxidase was performed by incubating sections with 10% hydrogen peroxide in PBS for 10 minutes after being deparaffinization. The tissue sections were heated at 95°C with sodium citrate buffer (10 mM, pH = 6) for 30 min for heat-induced antigen retrieval. Then the slides were washed with PBS, and sections were incubated with primary antibodies for 50 minutes at room temperature (RT). After two times washing with PBS, the slides were immersed with secondary antibodies (MAD-000237Q) for 45 min at RT. The slides were washed two times in PBS again, and incubated with the chromogen 3,3′-diaminobenzidine (DAB) for 10 min at the same temperature and washed in tap water. Mayer’s hematoxylin dye was utilized to stain the nuclei. Then, the slides were mounted and examined under a light microscope (Olympus, Germany).

Vimentin is a cytoplasmic protein that is used as a marker for human cells with mesenchymal origin recognition, predominantly found in ovarian stroma and GCs within growing follicles. Ki67 a nuclear antigen, was used to detect proliferating cells. CD45 (*Ptprc)* is an inflammatory marker, that was used to detect inflammatory cells, such as leukocytes, macrophages, and T lymphocytes. CD45 can confirm the presence of immune responses against the xenograft.

Based on the Smitz and Cortvrindt classification, the isolated ovarian follicles were allocated in different groups as follows: Primordial follicles were detected by the presence of an oocyte with a single flattened layer of granulosa cells (GCs); primary follicles were characterized by a single layer of cuboidal GCs (30–60µm) surrounding the oocyte; and secondary follicles were identified by two or more layers of cuboidal GCs (80–150µm) [42].

### RNA extraction and real-time PCR

In this research, three genes were investigated, including GDF9, which was related to human follicles and Vegf and Cd45 for mouse genes (n = 30).

For RNA extraction, all AO samples were washed with PBS, chopped, and transferred into a microtube. After adding Trizol and pipetting in 15 minutes, chloroform was added and the microtube was shaken. After 10 minutes, the samples were centrifuged (1200 xg) for 15 minutes at 4°C. The upper phase was transferred into a new microtube and an equal volume of isopropanol was added with gentle pipetting. The samples were left overnight at −20°C. In the next step, the sample was centrifuged at 1200 xg for 15 minutes at 4°C. Isopropanol was removed and 500 µl 70% ethanol was added. The samples were centrifuged at 7500 × g for 8 minutes at 4°C. Ethanol was removed and 25 µl RNase free H_2_O was added. The extracted RNA was utilized to make cDNA using RT, buffer, random primer, oligo dT, Ribolock, dNTP, and H2O. In the final step, SYBR Green, forward and reverse primers, and H2O were added. All primers are listed in Table 1.

**Table 1:** primer sequences for Real-time PCR.

Gene	Accession number	Primer	Product size (bp)
GAPDH	NM_001256799.3	F: 5’ GAAATCCCATCACCATCTTCC 3’ R: 5’ GGCTGTTGTCATACTTCTCAT 3’	219
GDF9	NM_001288828.3	F: 5’ GAA GTC TGT CTG CCT ATC CTG 3’ R: 5’ ACG GTC TTG GCA CTG AGG 3’	369
Gapdh	NM_001411842.1	F: 5’ GACTTCAACAGCAACTCCCAC 3’ R: 5’ TCCACCACCCTGTTGCTGTA 3’	125
Vegf	NM_009505.4	F: 5’ CTACTGCCGTCCGATTGAG 3’ R: 5’ GCTTTGTTCTGTCTTTCTTTGG 3’	266
CD45(Ptprc)	NM_001111316.3	F: 5’ CTGGTGTTTCTGATTATTGTGAC 3’ R: 5’ TTTCATCATCCCTTTCAACGA 3’	127

### Hormonal analysis

One week after xenotransplantation, mice blood serum was collected and used for hormonal analysis by electro chemiluminescent immunoassay (ECLIA) method. Blood samples were taken from the heart of xenografted mice and kept for 60 minutes at 4°C. Then, the samples were centrifuged at 4000 rpm for 20 minutes. Afterwards, the uppermost medium containing the blood serum was separated for human estrogen and progesterone analysis (5 replicates for each group).

### Statistical analysis

Statistical analysis was performed using Prism software (version 9.0, GraphPad Software, San Diego, CA, USA). Normality of the data was assessed using the Shapiro-Wilk test. Parametric data were analyzed using one-way ANOVA followed by t-tests for group comparisons. Results are presented as mean ± SD. A *P*-value less than 0.05 (denoted by *) was considered statistically significant. Additional significance levels are indicated by **, ***, and **** corresponding to *P* < 0.01, *P* < 0.001, and *P* < 0.0001, respectively.

## Results

### Assessment of decellularized WJ

**Fig 2:**
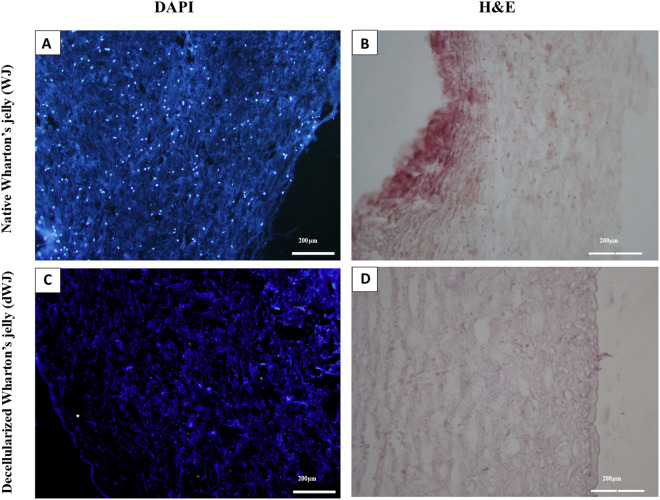
Wharton’s Jelly assessments after decellularization. A and **C)** DAPI staining shows that there are no residual cells in decellularized Wharton’s Jelly (dWJ). Otherwise, the bright nuclei can be observed in Native Wharton’s Jelly (WJ). B and **D)** Hematoxylin and eosin (H&E) staining confirmed the decellularization (*p* value < 0.05).

The decellularization results are shown in Fig 2. The WJ decellularization was confirmed by H&E staining. There are no cell residues in histological assessments including DAPI staining (Fig.2C) and H&E staining (Fig 2D). DNA content assessment was done in our previous study. It showed a significant difference between native and decellularized WJ samples (*p* value < 0.05).

### Histological evaluations

**Fig 3:**
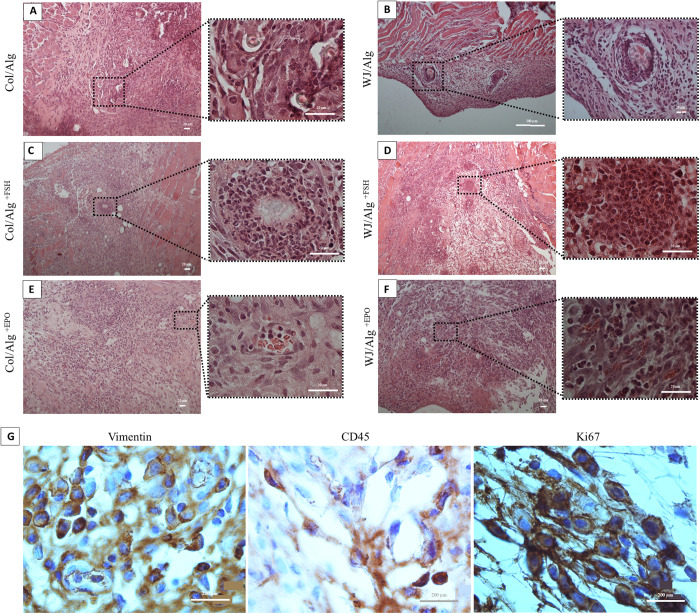
Histological and immunohistochemical assessments. **A and B)** H&E staining shows that both hydrogels can maintain follicles one week after xenotransplantation. However, Wharton’s Jelly/Alginate (WJ/Alg) can improve the follicle size compared to Collagen/Alginate (Col/Alg) **(B)**. **C and D)** FSH injection can increase granulosa cell (GC) proliferation after one week. This proliferation is higher in the WJ/Alg^+FSH^ group compared to the Col/Alg^+FSH^ group (507.6 ± 134.1 vs 1444 ± 493.6). **E and F)** Erythropoietin injection can enhance angiogenesis, especially in the Col/Alg group **(E)**. **G)** Immunohistochemical staining of Vimentin and Ki67 confirms the presence of human cells and proliferation. Also, CD45 is expressed in cells of the mouse immune system which may play a role in follicle deletion.

All of the groups were obtained 1 week after xenotransplantation. H&E evaluations showed that Col/Alg groups can maintain some of the follicles after 1 week (Fig 3A). Although the number of residual follicles in the Col/Alg group was more than in WJ/Alg group(7.000 ± 2.121 vs 2.200 ± 1.924). On the contrary, WJ/Alg can help to increase follicle size in addition to preserving the follicle structure (Fig 3B). Also, contrary to expectations, FSH injection had no improvement in follicle maintenance, but it can help GCs differentiation in both groups (Fig 3C,3D). Although the GC differentiation was more in dWJ/Alg^+FSH^ group than Col/Alg^+FSH^ group, oocytes were diminished in both groups (507.6 ± 134.1 vs 1444 ± 493.6). On the other hand, examining the transplanted region in the H&E staining at a glance confirmed that erythropoietin had an angiogenic effect and improved vascularization system in artificial ovaries in both WJ/Alg or Col/Alg constructs (Fig 3E,3F). Despite this event, EPO injection could not maintain xenotransplanted follicles either. It should be noted that most of the follicles were primordial (p-value <0.0001; 34.5 ± 1.157).

In immunofluorescence staining, the presence of human, proliferative, and inflammatory cells was confirmed by vimentin, Ki67, and CD45 antibodies, respectively (Fig 3G). Positive cells in the vimentin group confirm the presence of human cell proliferation. The number of these cells in the WJ/Alg group was more than the other groups (16.6/120 m^2^ in dWJ/Alg and 9.0/120 m^2^ in Col/Alg). Although there is no significant differences between both groups (p value > 0.05). Maybe increasing the number of replications can show a clear difference.

### Real-time PCR analysis

**Fig 4:**
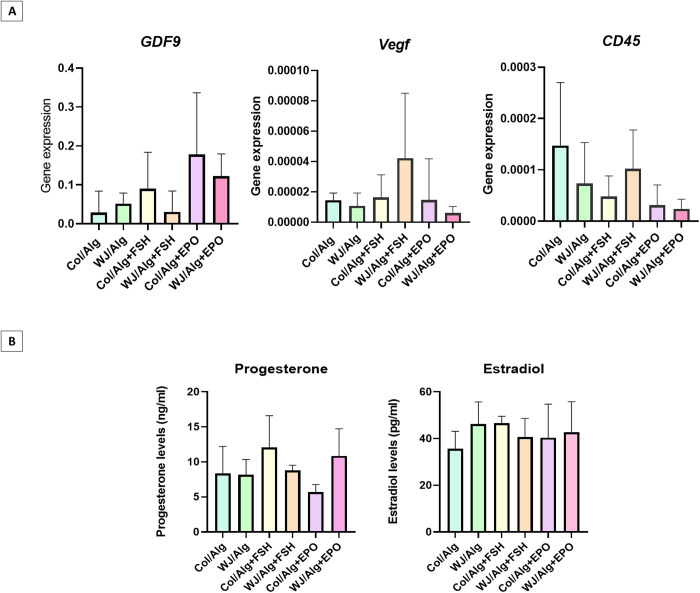
Gene expression and Hormonal assessments: A) One growth factor for human ovarian follicles (GDF9) and two genes in mouse tissue, including the vascular gene (Vegf) and the inflammatory gene (CD45; Ptprc) were examined. There are no significant differences among the groups. **B)** There are no significant differences between the groups in estradiol and progesterone hormone levels (p value > 0.05).

The human gene GDF9 and the mouse genes Vegf and Cd45 were evaluated. As shown in Fig 4A, there are no significant differences among all groups (p value > 0.05).

### Hormonal analysis

The hormonal analysis was done on mouse blood serum of all groups (n = 30), and the results are shown in Fig 4B. There is no significant difference in human estradiol and progesterone levels between the WJ/Alg or Col/Alg, dWJ/Alg^+FSH^, Col/Alg^+FSH^, dWJ/Alg^+EPO^ and Col/Alg^+EPO^ groups (p value > 0.05).

## Discussion

This study compared two different natural hydrogels, including WJ/Alg and Col/Alg as human artificial ovaries to support human ovarian follicles after 1 week xenotransplantation in mice (Fig 1). FSH and Erythropoietin injections were administered to promote hormonal support and induce the angiogenic factors in the respective groups (WJ/Alg^+FSH^, Col/Alg^+FSH^, WJ/Alg^+EPO^ and Col/Alg^+EPO^). Histological assessments generally showed that WJ/Alg can maintain the follicles and help to increase their size compared to the other groups. In addition, there were no significant differences in gene expression and hormonal analysis between all groups.

For the first time, Wharton’s Jelly was utilized as an AO in Zand et al. study to aid mouse ovarian isolated follicles autotransplantation [29]. Our previous study also showed that this beneficial hydrogel can support human ovarian follicles compared to the common Alg hydrogel (unpublished data). Since WJ hydrogel does not have enough mechanical consistency to handle well during experiment and xenotransplantation, it is better to combine it with portable hydrogel like alginate to increase WJH stiffness [29]. Some studies showed that alginate can improve mouse ovarian follicle growth [43], but there are no reports of using alginate to make human artificial ovary construction. Certain properties of alginate may play a role; for example, its stiffness can impair follicle growth [44], but some alterations in Alg hydrogel can make it more suitable for follicle growth [43]. Our experiments demonstrate that Wharton’s Jelly hydrogel as a natural bioengineered material in combination with Alg can address this problem. WJ/Alg combination can maintain human ovarian follicles and help their growth in the xenotransplantation system, like what was seen in Zand et al. article for mouse follicles [29]. In fact, the WJ Extracellular matrix (ECM) contains collagen, hyaluronic acid, and glycosaminoglycans, as well as many growth factors such as bFGF, EGF, PDGF, and TGF-β [28,45]. All of these factors can synergistically enhance Alg hydrogel bioactivity and improve follicle growth [29]. The wonderful capacity of the WJ Extracellular matrix is figured out in different studies. For the example, the decellularize WJ ECM was used as injectable hydrogel in many newest studies to treat various diseases including intervertebral disc degeneration [46], healing and cartilage protection [47], and cardiac failure [48]. Our results confirm that WJ/Alg hydrogel has the proper ability to support human ovarian follicles growth. However more investigations need to improve this AO.

On the other hand, the Collagen/Alginate group can preserve human ovarian follicles after 1-week xenotransplantation. Collagen is a natural biomaterial and the fundamental component of ECM that can crosslink with other materials for bioengineering purposes [22]. This scaffold was more used for 3D culture of mouse [25] or human ovarian follicles [49]. it was first time used as an artificial ovary to mouse ovarian follicle transplantation for 21 days by Telfer et al. [6]. In this study, mouse ovarian follicles were embedded in collagen hydrogel without being combined with other materials, and antral follicles were achieved. In addition, collagen alongside adipose-derived stem cells can improve premature ovarian insufficiency [50]. It should be mentioned that collagen was not obtained for human ovarian follicle transplantation. On the other side, in our experiment, collagen was combined of alginate to have a same condition as WJ/Alg hydrogel. Our result shows that collagen did not work efficiently to increase human ovarian follicles size as well as Wharton’s Jelly hydrogel. Using just Collagen/Alginate hydrogel for human ovarian follicle xenotransplantation may produce different results. Additionally, collagen concentration may have a role in this issue. In the current experiment, we used 4% collagen. Studying different concentrations of collagen can help to choose the right option to support human follicle growth. On the other side, collagen is one of the ingredients in Wharton’s Jelly. As a result, when WJ was used as an artificial ovary, it actually placed collagen properties in itself along with other functional materials like hyaluronan, and polysaccharide proteins [51]. WJ has at least three kinds of collagen including collagen I, III, and IV. Most of these collagens are insoluble in acidic solutions and neutral salts [51].

Our results show that FSH can increase GC proliferation in both groups (Fig 3C,3D; 507.6 ± 134 in the Col/Alg group vs 1444 ± 493.6 in the WJ/Alg group). However, they cannot play a role in oocyte survival and development and this is different from Dolmans et al.’s report [15]. They injected 7.5 IU FSH during the last two weeks of transplantation and achieved human antral follicles. There are some differences in the two experiments such as the type and number of transplanted follicles, the kind of hydrogel, mouse model, transplantation site, and graft duration. Although, in our current study, the Real-time PCR and hormonal data did not show the difference between all groups. GC proliferation can be seen clearly. It seems that FSH concentration adjustment can play a more positive role in follicle maintenance and growth.

On the other side, good vascularization has a key role in bioengineered applications [52]. Meanwhile, Erythropoietin injection could not maintain follicles in AO made by Col/Alg or WJ/Alg, increasing the blood vessels can be observed in histological staining (Fig 3E,3F). Erythropoietin is a kind of glycoprotein cytokine that plays a role in controlling erythropoiesis and act as an erythrocyte precursor in the bone marrow [53]. It also can be used for bioengineering purposes [54]. The EPO measurement was determined based on other studies [55,56]. Checking various doses of EPO can help select an optimum dose and improve the outcome of follicle transplantation.

Despite all efforts, there are some suggestions to improve the results. The current experiment was performed on NMRI mice. Using SCID mice can decrease immune rejection in grafted sites, especially in xenotransplanted AO. Checking different concentrations of Erythropoietin and FSH hormone adjustment can boost the AO ability to support human artificial ovaries and help achieve the end stage of follicles.

It should be mentioned, there are several limitations in this research, the most important being the lack of access to SCID mice. This limitation may significantly contribute to transplant rejection and follicle loss. Additionally, hormonal therapy may affect the ovaries of transgender individuals. Therefore, the number and quality of isolated follicles could be reduced. Overcoming these obstacles can help to improve the outcome of the research.

## Conclusion

Based on our results, although the combination of Col/Alg can maintain follicles in their primordial stage, Wharton’s Jelly/Alginate mixture has strong potential in supporting human ovarian follicle development during xenotransplantation. The use of this substance in the manufacture of artificial ovaries can play an important role in the fertility of the target groups.
